# The other immune landscape of SARS-CoV-2 evolution

**DOI:** 10.1073/pnas.2629158123

**Published:** 2026-09-28

**Authors:** Mahan Ghafari

**Affiliations:** ^a^ Department of Biology, University of Oxford, Oxford OX1 3EL, United Kingdom; ^b^ Pandemic Sciences Institute, Nuffield Department of Medicine, University of Oxford, Oxford OX3 7DQ, United Kingdom

What does SARS-CoV-2 adapt to as it circulates in the human population for sustained periods of time? Most answers, particularly after late 2021 with the emergence of Omicron, have focused on antibody escape. Successive variants have changed their spike protein and gained access to many individuals with preexisting immunity from vaccination and infection. This is the most visible, and perhaps easiest to quantify, part of SARS-CoV-2 evolution, but it does not explain the full picture. Infected hosts also differ along another axis, by molecules through which their T cells see the virus. And unlike the antibody histories, this variation is inherited and has changed very little over the course of the pandemic.

In this issue of PNAS, Riumina et al. show that variation in human leukocyte antigen (HLA) genes has left a measurable mark on SARS-CoV-2 evolution (1). Their study concerns HLA class I molecules, which display short viral peptides on the surface of infected cells. CD8+ T cells inspect these peptide–HLA complexes and can kill cells showing a recognizable target. A virus mutation can disrupt this process by weakening peptide binding to HLA or by preventing a T-cell receptor (TCR) from recognizing the complex.

The authors collected 1,450 experimentally supported CD8+ T-cell epitopes from IEDB (2), covering 63% of the virus proteome, and predicted how observed amino acid changes in the SARS-CoV-2 evolutionary tree altered binding to the relevant HLA molecules. Across mutations in the known epitopes, 35% of the lineage-defining mutations for variants of concern and 39% of the mutations arising within post-Delta and post-Omicron lineages were predicted to impair HLA binding. These relatively high proportions are suggestive of selection but are not convincing enough on their own. T-cell epitopes cover much of the proteome and some mutations can increase virus fitness for reasons unrelated to HLA-mediated immunity.

The stronger evidence comes from asking which HLA molecules the mutations evade. HLA frequencies vary greatly between different human populations. If escape from a given HLA molecule benefits the virus, mutations that disrupt its binding should do better where the HLA is common in the population. This is what Riumina et al. find. The most frequent binding-loss mutations associated with common HLA alleles were about 1.7 to 2.0 times as frequent as those associated with rare alleles. Many mutations were also more frequent in countries where their cognate HLA was more common. But the matched synonymous mutations did not show the same relationship. Therefore the geographical variation in human genetics can act as a natural experiment for changing the selective environment of the virus mutation.

**Fig. 1.**
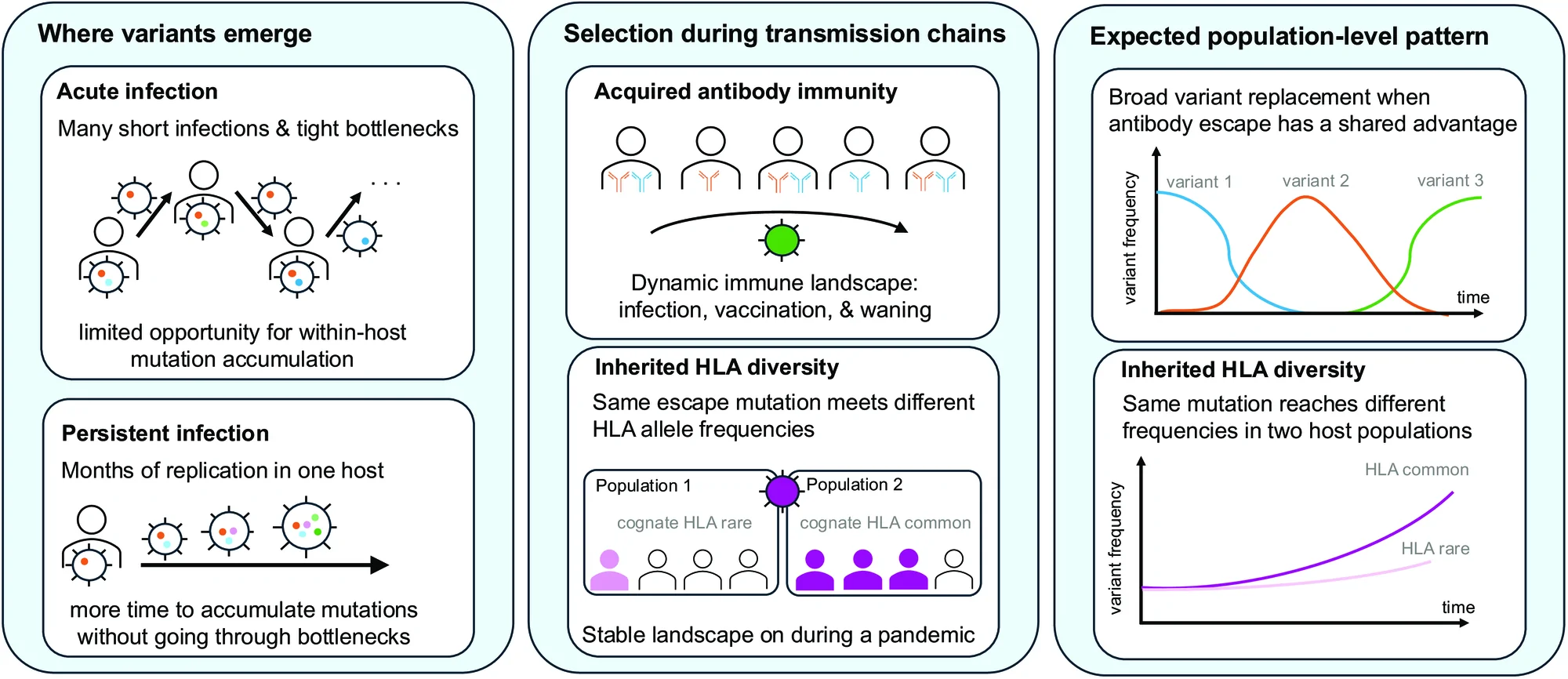
From variant emergence to population-level immune selection. Acute infections provide a continuous supply of stepwise evolutionary change and persistent infections may occasionally allow larger constellation of mutations to accumulate before onward spread. Antibody escape acts within a broadly shared but changing immune landscape and can contribute to successive variant replacements. In contrast, selection for HLA-mediated T-cell escape depends on stable geographic differences in cognate HLA allele frequency. Riumina et al. (1) found that escape mutations reached higher frequencies where their cognate HLA alleles were more common. They also showed that some mutations can disrupt TCR recognition despite increasing predicted peptide–HLA binding.

Antibody and HLA escape should therefore look different in genomic surveillance data (Fig. 1). Antibody immunity is acquired and reshaped by infection, vaccination, waning, immune imprinting, and virus evolution (3). Escape from a widely shared antibody response can give a lineage a large advantage and help replace the older lineage across many populations. HLA frequencies, on the other hand, are comparatively stable and geographically structured. Escape from one HLA response helps mainly in individuals who carry that allele. The same mutation may therefore increase in frequency when its cognate HLA is common, do little where it is rare, or hardly change at a global level.

In PNAS, Riumina et al. show that variation in human leukocyte antigen (HLA) genes has left a measurable mark on SARS-CoV-2 evolution (1).

A simple calculation gives a better sense of the strength of HLA selection. If a mutation has a 10% advantage in a matched immune environment which it encounters in 20% of transmissions, its average fitness advantage is about 2%, in the absence of other costs. That is enough to increase the frequency of that mutation over many transmission generations without necessarily producing a rapid selective sweep. A fitness cost also creates a threshold: the mutation may be favored where its cognate HLA is common but neutral or deleterious elsewhere. Most post-Delta and post-Omicron epitope variants studies by Riumina et al. were <~0.1% globally. Unlike lineage replacements associated with antibody escape, HLA adaptation may therefore consist of small, geographically structured changes in mutation frequencies rather than global replacements by one HLA-escape mutation.

The paper also shows why reduced peptide–HLA binding is not the whole story. Some mutations predicted to preserve or increase binding had the same geographic pattern expected for escape mutations. Riumina et al. proposed that these mutations disrupt TCR recognition instead. They tested seven variants of an ORF3a epitope with three TCRs. The three mutations supported by the geographic analysis showed significantly reduced TCR recognition. The other four mutations which did not have a geographic signal showed generally weaker effects. This was a small experiment but the observation is compelling as it shows that a population-level association predicted a molecular phenotype that peptide–HLA binding models had missed.

Where, then, does selection for HLA escape act: during acute infections, persistent infections, or both? Viral genetic diversity in the population must first arise within a host. Acute infections are individually poor settings for generating and transmitting much diversity because their short duration leaves little time for mutations to accumulate and, even then, only a small sample of the virus population survives the tight transmission bottleneck (4). But there are many more acute infections than persistent infections (5). Taken together, they make up a large continuous supply of single mutations that can accumulate step by step as a lineage circulates. Riumina et al.’s observation of a gradual increase in the average number of epitopes with lost HLA affinity per genome within Delta and Omicron lineages does not require an accelerated burst of evolution in a persistently infected host. HLA selection, like antibody selection, can act on mutations generated during ordinary transmission chains.

Persistent infections matter for a different reason. Months, or sometimes years, of replication without undergoing repeated transmission bottlenecks allow many mutations to accumulate in the same virus population before a descendant returns to wider circulation. Community genomic surveillance has found persistent infections with highly variable evolutionary rates, some of which are substantially accelerated (6). Earlier modeling suggested that this infection route is more likely to generate multimutation constellations on long stem branches that preceded the emergence of variants of concern (7). Persistent infections therefore do not need to explain the gradual accumulation of HLA escape within established viral lineages to remain important as a possible source of rare saltatory evolution of SARS-CoV-2.

Riumina et al. asked a related but different question: can selection during persistent infections alone account for the population-level HLA escape signal in their data? Across four different datasets, they found that mutations from persistent infections were no more strongly associated with common population HLA alleles than mutations accumulated during sustained transmission chains (1). This comparison is informative, but it does not clearly separate selection during persistent and acute infections. Since individual HLA types were not available for most persistent infection cases, the authors used country-level allele frequencies as proxies. Also, the phylogeny of the virus at the population level records viral ancestry rather than the clinical history of every host through which a lineage passed. A mutation generated during an unrecognized persistent infection can enter the wider population and later be counted among the mutations during sustained transmission chains. The most direct conclusion is therefore that population-level HLA association cannot be explained solely by selection during persistent infections.

Whatever its within-host origin, once a mutation enters the wider population, its fate depends on its net effect across many transmissions. For an HLA escape mutation, this includes the advantage it provides in hosts carrying the cognate allele, any costs it imposes in other hosts, and how often the virus encounters that allele. In this context, the connection between population-specific HLA selection and phylogenetic estimates of mutational fitness effects is particularly interesting (8). Bloom and Neher estimated the average between-host fitness effect of individual SARS-CoV-2 mutations by comparing how often a mutation arises independently on the phylogeny with how often it is expected to arise under a neutral baseline. In the community surveillance study, mutations found in persistent infections, particularly those found in more than one infection, tended to have higher between-host fitness effects than other background mutations (5, 6). This suggests that at least some mutations selected or tolerated during persistent infections can also spread successfully after transmission. It does not, however, directly show that persistent infections generated the HLA association that Riumina et al. found.

Riumina et al. used the number of independent origins of a mutation as a check on their analysis to see if they can still recover the association between HLA escape mutations and cognate HLA frequency once they replaced raw mutation frequencies with the number of independent origins. And they did. This makes hitchhiking within a successful lineage much less likely to explain the HLA association. However, independent recurrence of a mutation is still only a proxy for its fitness effect because different mutations are expected to arise at different rates. Bloom and Neher’s framework accounts for this by comparing the observed and expected number of origins of a mutation on a phylogeny. A potential next step would be to estimate these fitness effects across different host populations and ask whether they vary with the local frequency of the cognate HLA allele. Laboratory measurements, including deep mutational scanning of immune escape, could be considered alongside HLA frequencies. Such an analysis could help partition a mutation’s fitness effect into various components associated with a phenotype, including a component that depends on the HLA composition of the host population.

There is of course precedent for such host-dependent virus evolutionary dynamics in other viruses. HIV-1 repeatedly acquires cytotoxic T-cell escape mutations in people with particular HLA alleles and population adaptation follows geographic HLA frequencies (9). Some escape mutations revert after transmission to hosts lacking the selecting allele because their costs then outweigh their benefits (10). HLA-associated adaptation has also been observed in hepatitis C virus (11). SARS-CoV-2 differs from HIV-1 and hepatitis C virus in infection duration and transmission, but the same basic principle still applies that the effect of a mutation partly depends on the host genotype it encounters.

Riumina et al. do not replace antibody escape as a major contributor to SARS-CoV-2 evolution. They add another component of the virus fitness landscape, one that is inherited and stable over timespans of a pandemic and uneven across geography. The next challenge is to test whether measurements in defined HLA and TCR systems can predict the effects of individual escape mutations on growth rates across populations. If they can, models of SARS-CoV-2 evolution will need to include not just the virus and what human immune systems have seen but the human genetic variation through which the virus moves.
